# Synergistic Model of Cardiac Function with a Heart Assist Device

**DOI:** 10.3390/bioengineering7010001

**Published:** 2019-12-19

**Authors:** Eun-jin Kim, Massimo Capoccia

**Affiliations:** 1 Fluid and Complex Systems Research Centre, Coventry University, Coventry CV1 2TT, UK; 2 School of Mathematics and Statistics, University of Sheffield, Sheffield S3 7RH, UK; 3 Aortic and Cardiac Surgery, Royal Brompton Hospital, Sydney Street, Chelsea, London SW3 6NP, UK; capoccia@doctors.org.uk

**Keywords:** cardiac function, modeling, self-organization, lumped-parameter model, left ventricular pressure–volume relation, LVAD

## Abstract

The breakdown of cardiac self-organization leads to heart diseases and failure, the number one cause of death worldwide. The left ventricular pressure–volume relation plays a key role in the diagnosis and treatment of heart diseases. Lumped-parameter models combined with pressure–volume loop analysis are very effective in simulating clinical scenarios with a view to treatment optimization and outcome prediction. Unfortunately, often invoked in this analysis is the traditional, time-varying elastance concept, in which the ratio of the ventricular pressure to its volume is prescribed by a periodic function of time, instead of being calculated consistently according to the change in feedback mechanisms (e.g., the lack or breakdown of self-organization) in heart diseases. Therefore, the application of the time-varying elastance for the analysis of left ventricular assist device (LVAD)–heart interactions has been questioned. We propose a paradigm shift from the time-varying elastance concept to a synergistic model of cardiac function by integrating the mechanical, electric, and chemical activity on microscale sarcomere and macroscale heart levels and investigating the effect of an axial rotary pump on a failing heart. We show that our synergistic model works better than the time-varying elastance model in reproducing LVAD–heart interactions with sufficient accuracy to describe the left ventricular pressure–volume relation.

## 1. Introduction

Self-organization is a novel phenomenon in complex systems, whereby a macroscopic order emerges and is maintained in the midst of complexity [1]. One remarkable manifestation of self-organization is homeostasis in biosystems, known to be absolutely critical to the sustainability of living organisms. As one of most beautiful and important examples of self-organized biosystems [2], the human heart is a mechanical pump acting as a main force driving blood throughout the body via the circulatory system, supplying oxygen and nutrients to the tissues, and removing carbon dioxide and other waste products. The action of this mechanical pump is tightly regulated and is self-organized by feedback mechanisms among mechanical, electric, and chemical activities. Furthermore, cardiac function is self-organized and synchronized across scales, from microscale sarcomere to macroscale organ levels. Unfortunately, the breakdown of self-organization leads to heart diseases and failure, the number one cause of death worldwide. 

Mechanical circulatory support with rotary blood pumps, such as HeartMate II (an axial pump manufactured by Thoratec Corporation, Pleasanton, CA, USA) [3] and HeartWare ventricular assist device (HVAD: a centrifugal pump manufactured by Medtronic, Minneapolis, MN, USA) [4], is a recognized treatment for advanced heart failure patients, either as a bridge to transplant or recovery or ultimately as a long-term solution for noneligible candidates (destination therapy). Although very successful, the insertion of a left ventricular assist device (LVAD) remains associated with complications such as pump thrombosis, stroke, bleeding, and right heart failure, which affect outcomes [5,6,7,8,9,10]. It is, thus, crucial to increase our understanding of feedback mechanisms and heart–LVAD interactions.

The single most powerful measure to understand heart function and differentiate normal from pathological heart, as well as to diagnose or treat different cardiac pathology, is the relation between left ventricular pressure and volume—the so-called P–V loop. In particular, pressure–volume relation applied to lumped-parameter representation of the cardiovascular system has important clinical applications [6,11], owing to great flexibility in simulating the hemodynamics of different cardiovascular conditions and therapeutic interventions at a low computational cost, facilitating a personalized therapy [11]. However, the traditional approach heavily relies on the time-varying elastance model [11,12,13,14,15,16], where the ratio of the pressure to the volume (minus the ventricular volume at zero pressure) is prescribed as a periodic (double-Hill) function of time for a given heart rate, varying between given $E_{max}$ (the maximum value of E) and minimum $E_{min}$ (the minimum value of E). Although the time-varying elastance model has been useful in understanding the pressure–volume relation in different types of mammals, it is empirically based on (almost) physiological data where the heart is regulated internally. Thus, its validity in extreme, pathological conditions (such as in [17] in a study on mice) is questionable. For instance, when heart rhythm is not regular but chaotic (as in the case of fibrillation), elastance will also be chaotic rather than a simple periodic function. This issue is particularly important for the treatment of a failing heart requiring the insertion of a LVAD. In particular, since the device is connected in parallel between the aorta and the left ventricle to relieve its load, it is very likely to disturb or at least alter the status of the heart. There has been indeed some evidence against the validity of the time-varying elastance model with an assist device [18].

To face this challenge, in this paper, we propose a synergistic model which couples mechanical, electric, and chemical activity on microscale sarcomere and macroscale heart levels and investigate the effect of an axial rotary pump (e.g., Heart Mate II) on a failing heart. Our focus is to find an alternative method that is readily available within the constraints of the clinical environment. Lumped-parameter models are well suited for this purpose. We, thus, develop a comparable lumped-parameter model that predicts a P–V loop and LVAD–heart interaction by evolving left ventricular pressure and volume simultaneously at the minimum computational cost, instead of using the time-varying elastance concept. 

Specifically, we propose the two types, which have the same mechanical or chemical and electric activity set up but different circulation models. depending on the coupling between the left ventricle and the systemic arterial circulation—the basic (extended) model without (with) coupling to the systemic arterial circulation. Our models consist of 9 or 12 ordinary differential equations, and thus require significantly less computational resources and time compared with more advanced models [19] governed by partial differential equations. In particular, [19] simulated left ventricular fluid–solid mechanics through the cardiac cycle under LVAD support. CircAdapt [20] is another example of advanced models incorporating electric and sarcomere dynamics, but a systematic study with LAVD seems lacking.

## 2. Materials and Methods 

### 2.1. Mechanical/Chemical Model

On a microscale, we utilize the Bestel–Clement–Sorine (BCS) model of the dynamics of the contractile element [21,22,23] to evolve the active stress $\tau_{c}$, stiffness $k_{c},$ strain $\varepsilon_{c}$, and its velocity $v_{c}=\frac{d\varepsilon_{c}}{dt}$. In the BCS model, the active force derives from the chemical activity (e.g., ATP) modeled by u (measured in s^−1^). The governing equations for $v_{c},\;\varepsilon_{c},\;\tau_{c}$, and $k_{c}$ are then as follows:

(1) $$\frac{dv_{c}}{dt}=-\chi v_{c}-\omega_{0}^{2}\varepsilon_{c}-a\tau_{c}d_{0}\left(\varepsilon_{c}\right)+b\left(\sqrt{\frac{V}{V_{0}}}-1\right),$$

(2) $$\frac{d\varepsilon_{c}}{dt}=v_{c},$$

(3) $$\frac{d\tau_{c}}{dt}=k_{c}v_{c}-\left(\alpha_{l}\left|v_{c}\right|+\left|u\right|\right)\tau_{c}+\sigma_{0}u\Theta \left(u\right),$$

(4) $$\frac{dk_{c}}{dt}=-\left(\alpha_{l}\left|v_{c}\right|+\left|u\right|\right)k_{c}+k_{0}u\Theta \left(u\right),$$

(5) $$d_{0}\left(\varepsilon_{c}\right)=\exp\left(-\beta_{0}\;{\left(\varepsilon_{c}\right)}^{2}\right).$$

Here, Θ(u) is a Heaviside function that takes the value of 1 for u > 0 or 0 otherwise. The evolution of $v_{c}$ in Equation (1) involves a damping force $\chi v_{c}$, a harmonic force $\omega_{0}^{2}\varepsilon_{c}$, an active force $a\tau_{c}d_{0}\left(\varepsilon_{c}\right)$, and a passive force b($\sqrt{V/V_{0}}$−1), where $\chi ,\;a,\;b,\alpha_{l}$ are positive constants. Here, $\omega_{0}^{}$ is a microscale, high-oscillation frequency. Equation (1) is based on a simplified model of a cylindrical heart with a constant height [23], where the left ventricular volume V is related to the strain ε as a square root $\varepsilon \propto \sqrt{V}$. In Equations (3) and (4), the term involving $\alpha_{l}\left|v_{c}\right|+\left|u\right|$ represents the deactivation of contractile force, while the term involving uΘ(u) represents its activation due to a chemical input u>0. Additionally, $d_{0}\left(\varepsilon_{c}\right)$ in Equations (1) and (5) represents the length–tension curve of the contractile element, which we model using a Gaussian function for simplicity. The form of Equations (1)–(4) is identical to the BCS model.

To further link microscale dynamics in Equations (1)–(5) to macroscale dynamics, we relate $\tau_{c}$ in Equations (1) and (3) to the left ventricular pressure $P_{V}$ [23]:

(6) $$P_{V}=\gamma \frac{V_{0}}{V}[d_{0}\left(\varepsilon_{c}\right)\tau_{c}+\sigma_{P}],$$

(7) $$\sigma_{P}=\frac{k_{2}}{k_{1}}[\exp(k_{1}\sqrt{V/V_{0}}-1)-1],$$

where $\sigma_{P}$ represents the passive stress, which is assumed to be exponential in Equation (7) for simplicity; $k_{1}$ and $k_{2}$ are non-negative parameters for the passive tension. In Equation (6), γ is a constant parameter proportional to the ratio of left ventricular wall thickness to its radius, and its physiological meaning is provided in [21]. For our purpose of coupling microscale and macroscale dynamics, it suffices to treat this constant as a parameter proportional to the above ratio and tune it.

### 2.2. Electric Activity Model

We now link the chemical activity u in Equations (3) and (4) to the electric activity. To this end, we adapt the forced Van der Pol [2] and FitzHugh–Nagumo [24] models for our convenience (the precise form is not particularly important, given the nonlinearity of the equations), as follows:

(8) $$\frac{dp}{dt}=0.1\left(q-p+\mu_{1}\tau_{c}\right),$$

(9) $$\frac{dq}{dt}=10q\left(1-q^{2}\right)-10{\left(2\pi \right)}^{2}p+\mu_{2}V\Theta \left(V-V_{0}\right)+10\cos\left(2\pi t\right),$$

(10) $$u=\alpha_{u}q,$$

where p and q are dimensionless slow and fast variables for the electric activity, respectively; 10cos(2πt) in Equation (9) represents the force with 1 Hz frequency, which we fix to model a normal heart with heart rate 1 Hz as a control case. Equation (10) represents the chemical activity u proportional to electric activity (q) with a proportional constant $\alpha_{u}>0$. Here, $\mu_{1}$ and $\mu_{2}$ in Equations (8) and (9) represent the mechano-electric feedback (MEF) [25,26,27,28,29]. The non-zero value of $\mu_{1}$ or $\mu_{2}$ introduces the coupling between (p,q) and $\tau_{c}$ or V. Specifically, $\mu_{1}$ mimics the effect of mechanical stress due to shortening during a contraction on the action potential and is motivated by [25]; $\mu_{2}$ models the effect of stretch (e.g., through a stretch-activated ion channel) [28]. Biologically, the values of $\mu_{1}$ and $\mu_{2}$ could be changed due to the over or under expression of an ion channel. The units of $\mu_{1}$ and $\mu_{2}$ are kpa^−1^ and (s·mL)^−1^, respectively. Since the role of MEF in a beating heart is implicit, we are uncertain about the absolute value or sign of $\mu_{1}$ and $\mu_{2}$ for a control case. However, we find that $\mu_{1}$ = 0.0024 kpa^−1^ gives a reasonable relaxation time (about 0.4~0.5 s for 1Hz heart rate). We, thus, choose $\mu_{1}$ = 0.0024 kpa^−1^ and $\mu_{2}$ = 0 for a control case. In the remainder of the paper, we omit the units of $\mu_{1}$ and $\mu_{2}$ for simplicity. 

### 2.3. Basic Circulation Model and Control Case

For the circulation model, at the simplest level, we ignore the dynamics of the systemic arterial circulation and propose the following basic model, which evolves the left ventricular volume V, aortic pressure m, and pump flow n through an axial rotary pump

(11) $$\frac{dV}{dt}=\frac{1}{R_{M}}\left(P_{R}-P_{V}\right)\Theta \left(P_{R}-P_{V}\right)-\frac{1}{R_{A}}\left(P_{V}-m\right)\Theta \left(P_{V}-m\right)-\delta_{p}n,$$

(12) $$\frac{dm}{dt}=-\frac{1}{C_{S}R_{c}}\left(m-m_{0}\right)+\frac{1}{C_{A}R_{A}}\left(P_{V}-m\right)\Theta \left(P_{V}-m\right)+\delta_{p}\frac{n}{C_{A}},$$

(13) $$\frac{dn}{dt}=\frac{\delta p}{L_{*}}[P_{V}-m-R_{*}n+\;\beta \omega^{2}].$$

In Equation (12), m is not coupled to the aortic flow but instead has a parameter $m_{0}$, which models a constant arterial pressure. In Equation (11), $P_{R}$ is the atrial pressure, and for our basic model, we take this as a constant value instead of treating it dynamically. Increasing $P_{R}$ and $m_{0}$ has the effect of increasing preload and afterload, respectively. Equations (11) and (13) are exactly the same as those in [14,15]. Here, $\delta_{p}$ is a parameter to denote the presence of a pump, where $\delta_{p}=1$ ($\delta_{p}=0$) represents the presence (absence) of a pump. $R_{c}$, $R_{M},\;R_{A}$, and $R_{*}$ are resistances, $C_{S}$ and $C_{A}$ are compliances, and $L_{*}$ is inertance. Equation (13) shows that the pump flow n through an axial pump is driven by $\beta \omega^{2}$, where β is the pump parameter and ω is the pump speed (not angular frequency), following the same notations as in [5,14,15]. Variables and parameters in Equations (1)–(12) are summarized in Table 1 and Table 2, respectively, together with their physiological meaning and their values. 

### 2.4. Extended Circulation Model and Control Case

**Figure 1:**
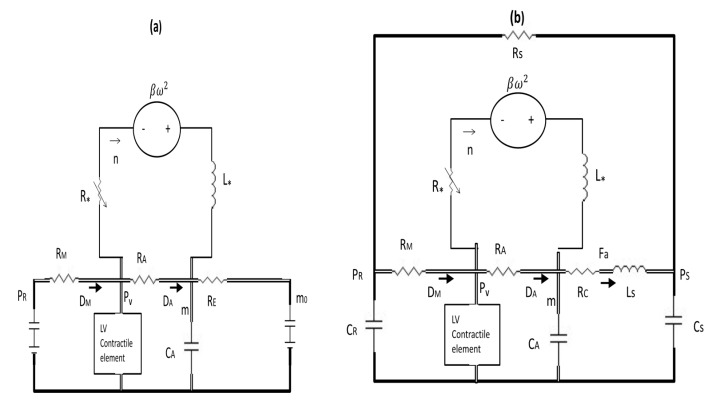
(**a**) The electric circuits for the basic model (**a**) and for the extended model (**b**). For the pump part, only total (effective) inductance $L_{*}$ and resistance $R_{*}$ are shown.

We extend our basic model by incorporating the dynamics of the systemic arterial circulation, specifically by treating the arterial and atrial pressures dynamically. We, thus, include the following evolution of Equations (16) and (17) for atrial pressure $P_{R}$ and arterial pressure $P_{S}$ based on the Windkessel model in [14,15], while generalizing Equation (12) to Equations (14) and (15) by including the aortic flow $F_{a}$:

(14) $$\frac{dm}{dt}=-\frac{1}{C_{A}}F_{a}+\frac{1}{C_{A}R_{A}}\left(P_{V}-m\right)\Theta \left(P_{V}-m\right)+\delta_{p}\frac{n}{C_{A}},$$

(15) $$\frac{dF_{a}}{dt}=\frac{m-P_{S}}{L_{S}}-\frac{R_{C}F_{a}}{L_{S}},$$

(16) $$\frac{dP_{R}}{dt}=\frac{-P_{R}+P_{S}}{C_{R}R_{S}}-\frac{1}{C_{R}R_{M}}\left(P_{R}-P_{V}\right)\Theta \left(P_{R}-P_{V}\right),$$

(17) $$\frac{dP_{S}}{dt}=\frac{P_{R}-P_{S}}{C_{S}R_{S}}+\frac{F_{a}}{C_{S}},$$

where $R_{S}$ is the systemic vascular resistance. Thus, for our extended model, we solve Equations (14)–(17), (1)–(11) and (13) using parameter values in Table 2. Equations (14)–(17) are identical to those in [14,15], with $P_{R}=x_{2},\;P_{S}=x_{3},$ $m=x_{4},F_{a}=x_{5}\;\left(V=x_{1},\;n=x_{6}\right),$ with the key difference that we calculate V and $P_{V}$ through the coupling to the microscale dynamics instead of using a prescribed time-varying elastance formula. Figure 1 shows the electric circuits for the basic model in Figure 1a and for the extended model in Figure 1b, where the detailed explanation is provided in the figure caption. 

Here, $D_{M}$ and $D_{A}$ represent diodes; (a) $R_{E}=R_{C}\frac{C_{S}}{C_{A}}>R_{C}$ and constant values of $P_{R}$ and $P_{S}=m_{0}$ are represented by batteries. To recover the basic model from the extended model, we let $R_{C}\to R_{E}$ and $P_{S}\to m_{0}$ in Equation (15) and obtain the steady solution $F_{a}=\frac{m-P_{S}}{R_{E}}=\frac{m-m_{0}}{R_{c}C_{S}}C_{A}.$ Substituting this in $\frac{1}{C_{A}}F_{a}$ in Equation (14) then gives $\frac{m-m_{0}}{R_{c}C_{S}}$ in Equation (12).

## 3. Results and Discussions

### 3.1. Control Case in Basic Model

**Figure 2:**
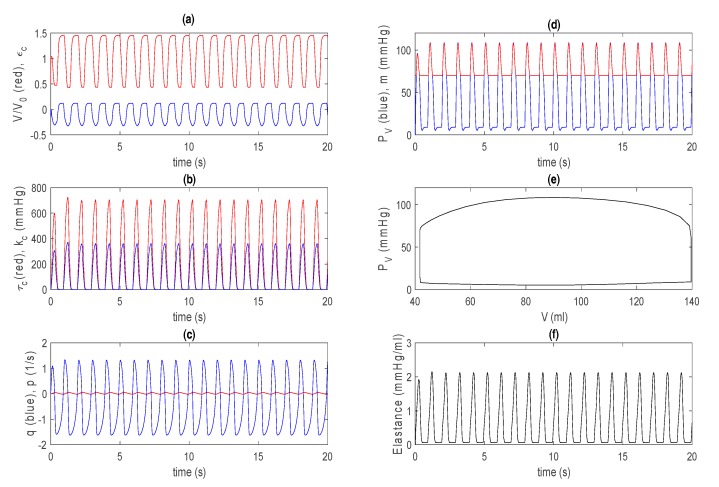
Control case for the basic model. (**a**) The $V/V_{0}$ (red) and $\varepsilon_{c}$ (blue) against time; (**b**) $\tau_{c}$ (red) and $k_{c}$ (blue) against time; (**c**) q (blue) and p (red) against time; (**d**) $P_{V}$ (blue) and m (red) against time; (**e**) $P_{V}$ against V; (**f**) elastance against time.

**Figure 3:**
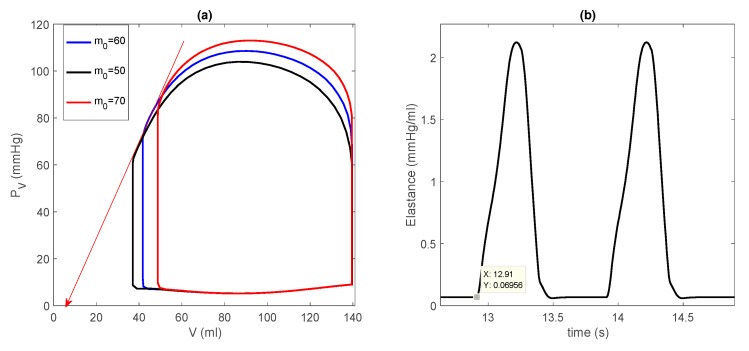
(**a**) Steady-state pressure–volume (P–V) loops for different afterloads: $m_{0}$ = 50, 60 and 70 mmHg; (**b**) E(t) in Figure 2f for the basic model. Note that a data cursor image shows $E_{min}$ on the y-axis.

As an example of a control case, we choose $P_{R}=9$ mmHg, $m_{0}$ = 70 mmHg, b=6000, γ = 0.6, $k_{2}$ = 14 kpa, $\alpha_{u}=5$, $V_{0}$ = 144/1.5 mL, $\mu_{1}=0.0024$, and $\mu_{2}=0$. The initial left ventricular volume is taken as V(t=0)= 0.5 $V_{0}$. For this basic model, solutions do not sensitively depend on other initial values, and settle into (almost) unique solutions quickly (after a few seconds). Figure 2 shows the time evolution of $V/V_{0}$ and $\varepsilon_{c}$ in Figure 2a, $\tau_{c}$ and $k_{c}$ in Figure 2b, q, p in Figure 2c, and $P_{V}$ and m in Figure 2d, where they all quickly move to a steady state from after only one or two transient oscillations. The initial transient is not shown in the P–V loop in Figure 2e. In Figure 2f, we show the elastance E(t)= $\frac{P_{V}}{V\left(t\right)-V_{*}}$ by using the volume at zero pressure $V_{*}$ ~ 7 mL, which is determined from the end systolic pressure–volume relation (ESPVR) and shown by a solid red line in Figure 3a for steady-state P–V loops obtained for different $m_{0}$ values. 

Figure 3a reveals that the larger the afterload $m_{0}$, the larger the end systolic volume (ESV) and the smaller the stroke volume (SV = EDV − ESV), where EDV is the end diastolic volume. In Figure 2f, the maximum and minimum values of E(t) are about 2.1 and 0.07 mmHg/mL, respectively, similar to those that are used for the time-varying elastance model for a normal heart [14,15]. Furthermore, the overall shape and time duration between contraction and relaxation are also qualitatively similar to those from the time-varying elastance model. This is shown in Figure 3b by magnifying Figure 2f. For larger values of $m_{0}$, the ESPVR is observed to be concave to the volume axis, consistent with the experimental results [17,30].

### 3.2. Control Case in Extended Model

**Figure 4:**
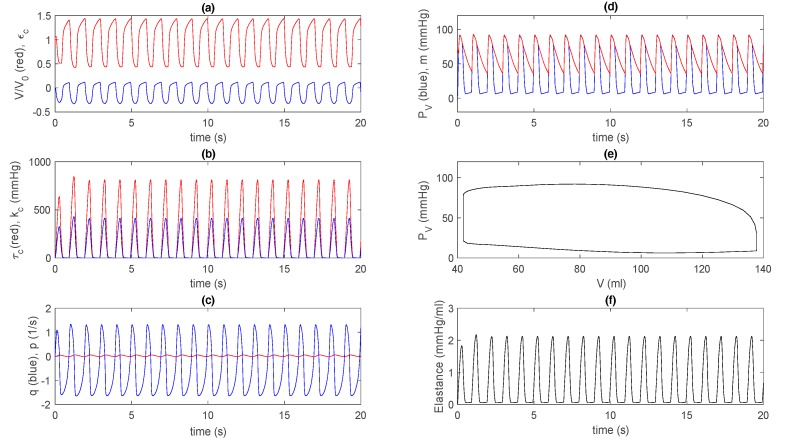
Control case for the extended model: (**a**) $V/V_{0}$ (red) and $\varepsilon_{c}$ (blue) against time; (**b**) $\tau_{c}$ (red) and $k_{c}$ (blue) against time; (**c**) q (blue) and p (red) against time; (**d**) $P_{V}$ (blue) and m (red) against time; (**e**) $P_{V}$ against V; (**f**) elastance against time.

**Figure 5:**
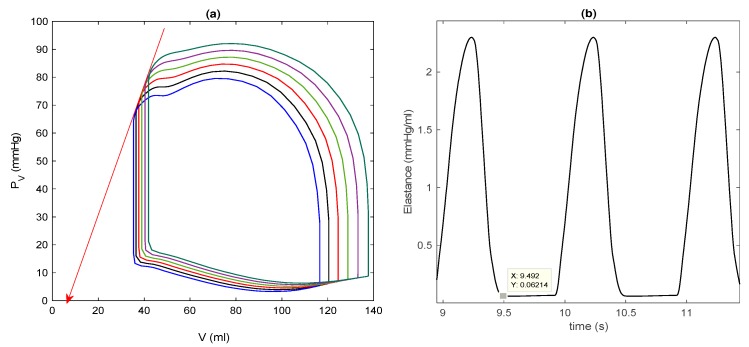
(**a**) Steady-state P–V loops for different afterloads: $V\left(0\right)=\left[0.4,\;0.5,\;0.6,\;0.7,\;0.8,\;0.9\right]V_{0}$ increasing to the right; (**b**) E(t) in Figure 4f for the extended model. Note that a data cursor image shows $E_{min}$ on the y-axis.

For the extended model, we use the same parameter values as in Figure 2 and Figure 3 (e.g., $k_{2}=14$ kpa, γ = 0.6, $V_{0}$ = 144/1.5 mL, etc.) and in Table 2. Since the circulation models for $P_{R}$, m, $P_{S}$, and $F_{a}$ are linear, solutions have a rather sensitive dependence on initial values in addition to parameter values. For Figure 4f, we use initial values $P_{R}\left(0\right)=$ 10 mmHg, $P_{S}$(0) = 70 mmHg, m(0)= 70 mmHg, $F_{a}$ = 90 mL/s, and V(t = 0) = 0.9 $V_{0}$. In Figure 4f, E(t) is calculated by determining the zero pressure volume $V_{*}$~7 mL (see Figure 5). Notably, the overall behaviour in Figure 4 is quite similar to that in Figure 2. In particular, the solutions go to a steady state form after only one or two transient oscillations in Figure 4a–d,f; The initial transient is not shown in the P–V loop in Figure 4e.

Figure 5 is equivalent to Figure 3 and shows different P–V loops in a steady state obtained for different initial conditions V(0)=[0.4,0.5,0.6,0.7,0.8,0.9] mL, as well as a detailed view of E(t) by magnifying Figure 4f. Interestingly, in Figure 5a, as V(0) increases, both EDV and ESV increase, similar to the effect of different preloads through interactions between preload and afterload, where an increase in SV leads to an increase in cardiac output, arterial pressure, and thus, afterload. For $V\left(0\right)>V_{0}$, we observe that ESPVR is concave to the volume axis, as in the basic model. Furthermore, Figure 5b is quite similar to Figure 3b, with similar maximum (around 2.1 mmHg/mL) and minimum (around 0.06 mmHg/mL) values. These results underscore that the basic model is a good approximation of the extended model for a control case.

### 3.3. Dilated Cardiomyopathy

Dilated cardiomyopathy is one of the main causes of heart failure, where the heart becomes enlarged and cannot pump blood effectively. This can result from left ventricular remodeling, such as a gradual increase in left ventricular EDV and ESV, wall thinning, and other causes. To model this pathological change in heart geometry, we increase the parameter values of $k_{2}$ and $V_{0}$ to $k_{2}=40$ kpa, $V_{0}$ = 144 mL while reducing γ to γ = 0.45 (these parameter values are in square brackets in Table 2) and fix all other parameter values.

#### 3.3.1. Effect of a Pump on Dilated Cardiomyopathy in Basic Model

**Figure 6:**
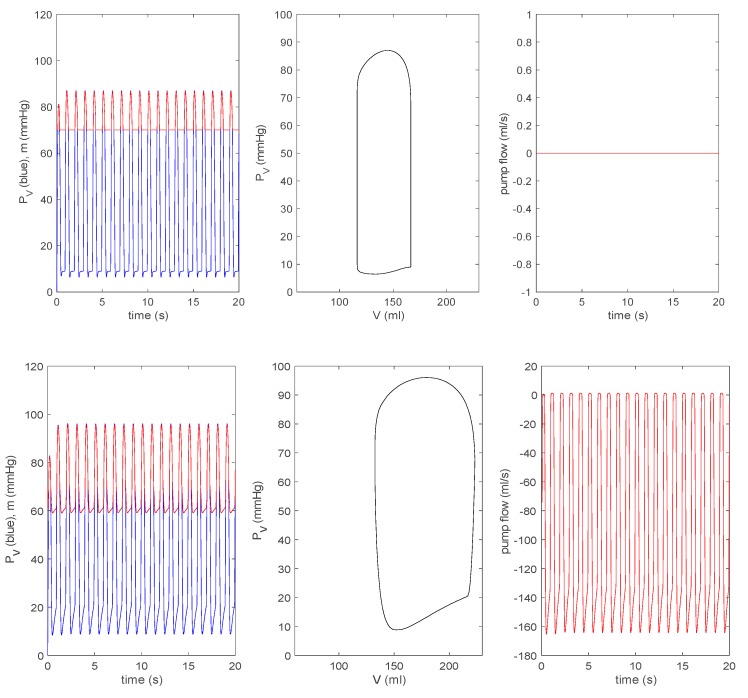

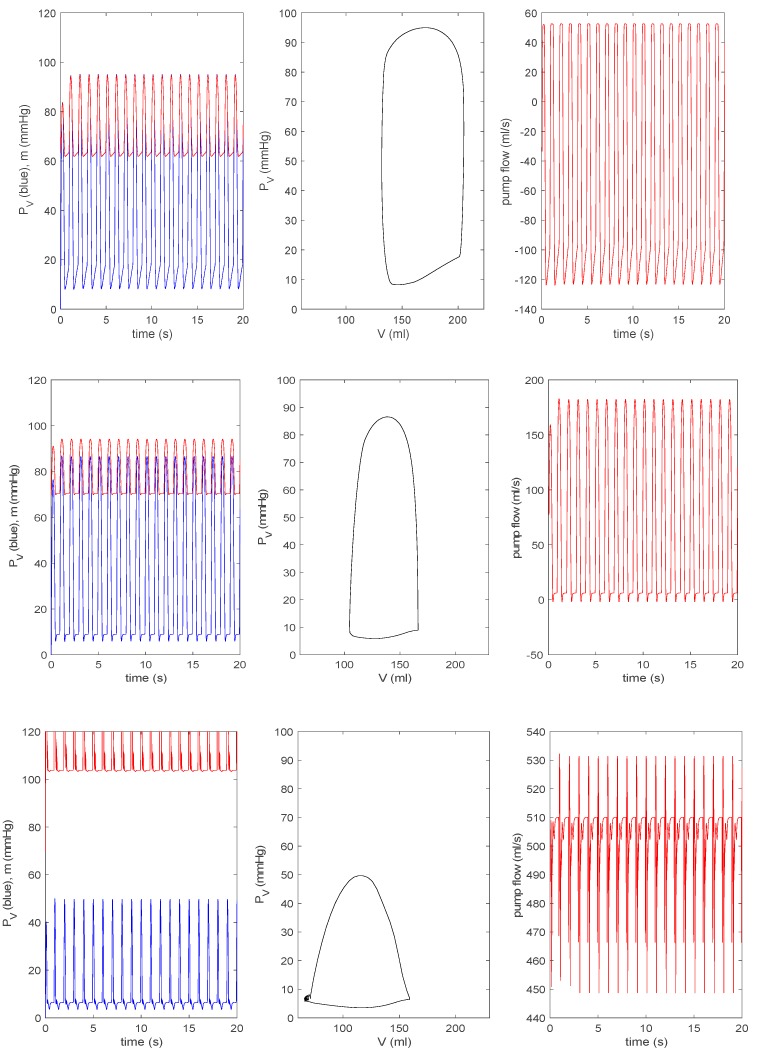
From top to bottom: $\delta_{p}=0,\;\omega =0,\;0.5\times 8000,\;8000,\;2\times 8000$ rpm, with $\delta_{p}=1$ for the basic model. In each row, the left panel represents $P_{V}$ (blue) and m (red) against time; the middle panel represents $P_{V}$ against V; the right panel represents the pump flow n against time.

For all other parameters, we use the same values as those used in Figure 2 and Figure 3. Figure 6 shows the time evolution of q (in blue) and p (in red) in the first column, a steady state P–V loop in the second column, and the evolution of pump flow n in the last column. The first row is for the case without a pump, where the zero pump flow simply means that there is no pump in this case. To see the effect of a pump, we choose $\delta_{p}=1$ in Equations (11)–(13) and vary the values of rotary pump speed (frequency) ω in Equation (13) as ω=[0,0.5,1,2]×8000 rpm, presenting results in the second to fifth rows in Figure 6. As ω increases, the P–V loop initially shifts to a larger V before shifting to a smaller V. The pump flow is negative (associated with an increase in V) for ω=0 and then becomes positive (with the decrease in V) as ω increases. This means that the pump speed needs to exceed the minimum value to work effectively (a pump would shut down at the speed below this minimum value). 

In clinical practice, 8000–10,000 rpm is the speed used for an axial rotary blood pump (HeartMate II) [10]. A speed < 8000 rpm is used when weaning off the LVAD and a certain protocol is followed, gradually decreasing the speed to 6000 rpm to allow ejection of the native heart and analyse its performance. In Figure 6, with the increase of ω, the left ventricular pressure $P_{V}$ eventually decreases, while aortic pressure m increases. This implies that in a failing heart, a pump will help increase the arterial pressure. In the bottom row, we see the decoupling between $P_{V}$ and m for ω=2×8000 rpm. These results are similar to those from the time-varying elastance model [14,15].

#### 3.3.2. Effect of a Pump on Dilated Cardiomyopathy in Extended Model

**Figure 7:**
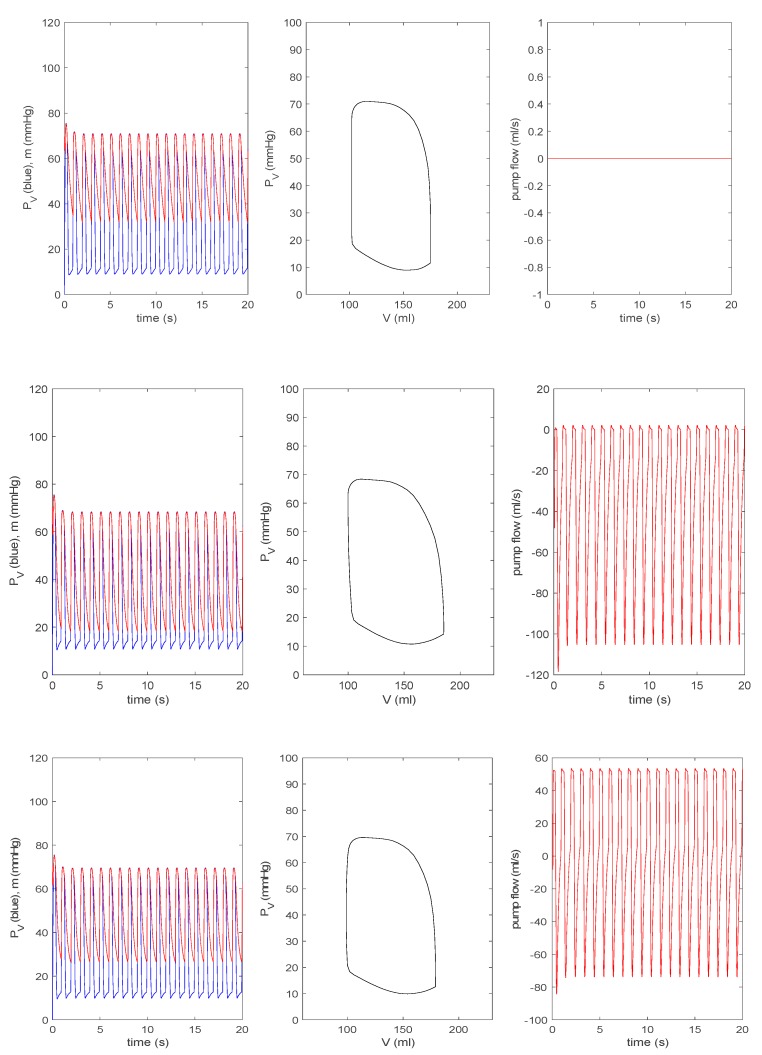

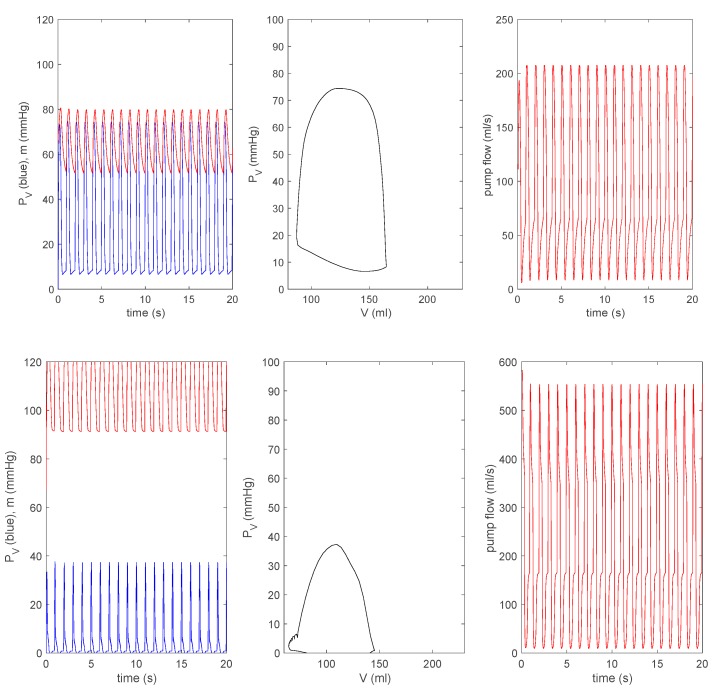
From the top to the bottom: $\delta_{p}=0,\;\omega =0,\;0.5\times 8000,\;8000,\;2\times 8000$ rpm, with $\delta_{p}=1$ for the extended model. In each row, the left panel represents $P_{V}$ (blue) and m (red) against time; the middle panel represents $P_{V}$ against V; the right panel represents the pump flow n against time.

**Figure 8:**
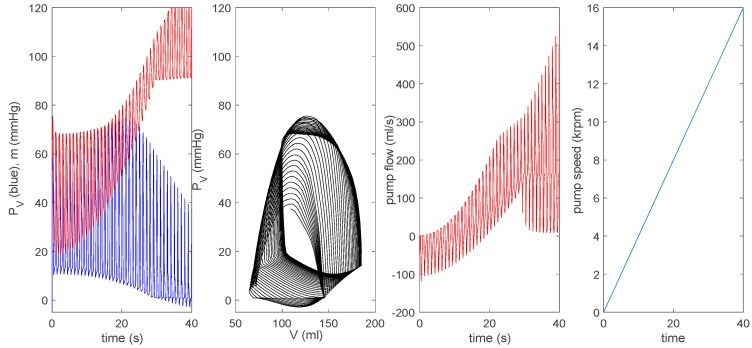

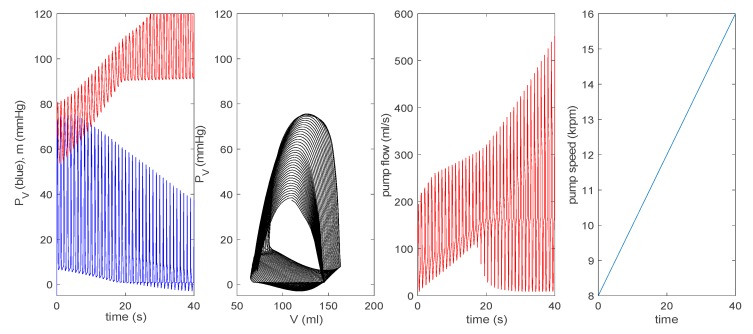
The top shows ω=400 t rpm and the bottom shows ω=8000+200 t rpm for the extended model.

For all other parameters, we use the same values as those used in Figure 4 and Figure 5. Figure 7 is equivalent to Figure 6. The top row is the case without a pump ($\delta_{p}=0$) for a pathological heart; the second to the bottom row in Figure 6 shows a pump ($\delta_{p}=1$) with a pump speed ω=[0,0.5,1,2]×8000 rpm. As in Figure 6, as ω increases, a P–V loop initially shifts to a larger V before shifting to a smaller V; the pump flow becomes negative (related to the increase in V) before becoming positive (associated with the decrease in V). With the increase of ω, $P_{V}\;\left(m\right)$ eventually decreases (increases). In the bottom row for ω=2×8000 rpm, $P_{V}$ and m decouple without any overlap between the two and $P_{V}$ contains negative pressure. Overall, by comparing Figure 6 and Figure 7, we observe the disparity in the behaviour of n, in particular for ω=2×8000 rpm. For a closer comparison with previous works [14,15], we make the pump speed a linearly increasing function of time as ω=400 t rpm and ω=8000+200 t rpm, and show the corresponding results together with the pump speed in the top and bottom panels of Figure 8, respectively. Overall, the behaviour of $P_{V}$, m, and n suddenly changes around ω ~ 12 krpm, with the formation of a large envelope of oscillations. This nonmonotonic behaviour corresponds to the onset of the suction in [14,15]. For larger ω, $P_{V}$ becomes negative. 

For a closer comparison with previous works [14,15], we make the pump speed a linearly increasing function of time as ω=400 t rpm and ω=8000+200 t rpm, and show the corresponding results together with the pump speed in the top and bottom panels of Figure 8, respectively. Overall, the behaviour of $P_{V}$, m, and n suddenly changes around ω ~ 12 krpm, with the formation of a large envelope of oscillations. This nonmonotonic behaviour corresponds to the onset of the suction in [14,15]. For larger ω, $P_{V}$ becomes negative. 

**Figure 9:**
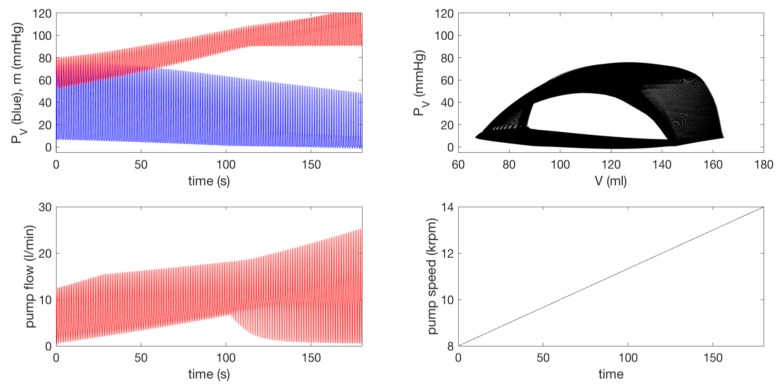
Here, ω=8000+100t/3 rpm for the extended model.

**Figure 10:**
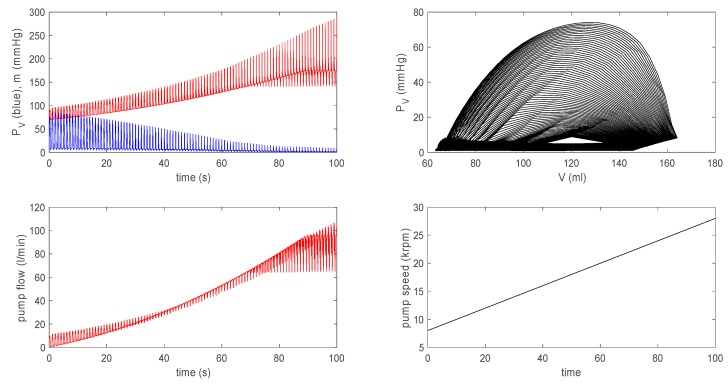
Here, ω=8000+200 t rpm for the basic model (compared with Figure 8 and Figure 9 for the extended model).

Further, to compare our results with in vivo animal data during rotary pump assistance (Figure 79.13 in [14], Figures 10 and 11 in [15]), we show results by using $\omega =8000+\frac{100t}{3}$ in Figure 9. In particular, the left bottom panel shows that the pump flow exhibits a large envelope of oscillations, while its mean value slowly increases over time; the onset of suction occurs at the pump speed ω≈12 krpm. This is very similar to the behaviour of the pump flow from in vivo data shown in Figure 79.13 in [14].

Here, a substantial oscillation envelope is seen, with the mean value increasing with time before the onset of suction, which occurs at a similar pump speed of ω=12.5 krpm. In comparison, the evolution of the pump flow from the time-varying elastance model shown in Figure 79.12 in [14] behaves differently; there is a significant reduction in the oscillation envelope without much increase in its mean value before the onset of suction, and the onset of suction takes place at a much greater pump speed of ω≈15.5 krpm. 

Therefore, our results are in much better agreement with in vivo data than the prediction from the time-varying elastance model in [14,15]. The agreement with in vivo data is also better in the extended model than the basic model, as aortic flow (its interaction with a pump) is not treated dynamically in the basic model (see Figure 10). These results highlight the importance of self-consistent treatment of the LVAD–heart interactions in simulating clinical scenarios, with a view to treatment optimization and outcome prediction. 

Finally, we note that the direct comparison of our results with those from more advanced (distributed) models with the LVAD support [19] is difficult. if not impossible, because of the use of different variables or parameters and the lack of a systematic study on these advanced models (due to high computational cost).

### 3.4. Summary

The pressure–volume relation plays a key role in cardiovascular modeling, and the traditional approach heavily relies on the time-varying elastance concept [5,11,12,13,14,15], in which the pressure to volume ratio is prescribed as a (double) periodic function of time. In particular, lumped-parameter models combined with pressure–volume loop analysis and time-varying elastance are very effective in simulating clinical scenarios, with a view to treatment optimization and outcome prediction [6,11]. However, the time-varying elastance concept has limited validity, as it cannot address cardiac self-organization and its breakdown, especially for a mechanically supported left ventricle, where its application for the analysis of LVAD–heart interactions has been questioned. Therefore, our aim was to develop a method that is at least as good as lumped-parameter modeling, but which enables us to investigate cardiac self-organization and the interaction between the cardiac system and LVAD. 

To this end, we developed a synergistic model that couples mechanical, electric, and chemical activity on microscale sarcomere and macroscale heart levels, and which predicted the pressure–volume relation of the left ventricle consistently. In a control case without LVAD, we showed that both basic and extended models reproduced the left ventricular pressure–volume relation, which is similar to what is obtained from the time-varying elastance model (e.g., comparing Figure 2, Figure 3, Figure 4 and Figure 5 with Figure 79.8b in [14]). 

We then considered a failing left ventricle by choosing parameter values to model dilated cardiomyopathy and investigated the interaction between a failing left ventricle and the circulation in the presence of a rotary pump by including the coupling between the aortic pressure and the pump flow in an axial rotary pump. For a sufficiently large pump speed, the stroke volume of the left ventricle increases, while its pressure and aortic pressure are decoupled with the increase (decrease) in the pressure of the aorta (left ventricle), similar to the finding from the time-varying elastance model. Furthermore, our extended model worked better than the time-varying elastance model in reproducing in vivo animal data with a rotary pump (see Figure 9), in particular in the overall shape (large envelope) of oscillations of the pump flow and the pump speed for the onset of suction, with a better agreement compared with the time-varying elastance model in [14,15].

## 4. Conclusions

We conclude that our synergistic model is a valid and better alternative to the time-varying elastance model considering that it addresses all the key aspects involved in terms of the overall P–V loop shape and the interaction between LVAD and circulation. In particular, it consistently incorporates the coupling between the mechanical and electric activity and can be used to explore MEF, which would otherwise have been impossible in real experiments or with the time-varying elastance model. The importance of self-consistent treatment of the LVAD–heart interactions was highlighted. Finally, we note that our model can also be extended to study the interaction between the left ventricle and atria [20], as well as the effect of LVAD on the right ventricle failure [31] at the simplest level, but yet is close to real life scenarios incorporating the interaction between the circulation, LVAD, and microscale dynamics. Future work will also explore the incorporation of central and peripheral extracorporeal membrane oxygenation (ECMO).

Limitation of our study: Given the simplicity of our lumped parameter model based on a set of ordinary differential equations, our model cannot inform us how our variables are distributed in space (e.g., waves), any consequences of their inhomogeneity, or the effect of boundaries. Furthermore, given the limited number of parameters, our model reproduces only a qualitatively similar shape of a P–V loop of an individual, but not its exact shape.

## Figures and Tables

**Table 1:** Summary of variables and meanings.

Variable	Physiological Meaning (Unit)
$v_{c}$	Velocity of the contractile element (s^−1^)
$\varepsilon_{c}$	Strain of the contractile element
$\tau_{c}$	Active tension of the contractile element (mmHg)
$k_{c}$	Stiffness of the contractile element (mmHg)
$\sigma_{P}$	Passive stress (mmHg)
u	Chemical activity (s^−1^)
p	Slow electric variable
q	Fast electric variable
$P_{V}$	Left ventricular pressure (mmHg)
V	Left ventricular volume (mL)
$P_{R}$	Atrial pressure (mmHg)
$P_{S}$	Arterial pressure (mmHg)
m	Aortic pressure (mmHg)
$m_{0}$	Arterial pressure parameter for the basic model (mmHg)
$F_{a}$	Aortic (total) flow (mL/s)
n	Pump flow (mL/s, mL/min)
$\delta_{p}$	Pump parameter (1 for pump, 0 for no pump)

Note that the parameter values for the contractile components ($\sigma_{o},\;k_{c},\;k_{1},\;k_{2}$) are based on [21,22,23], while those for resistance, compliance, inertance, pump, and are taken from [14,15]. The advantage of this basic model is that solutions are not too sensitive to initial conditions due to the nonlinearity of Equations (1)–(10) for mechanical and electric activity and the fixed value of $P_{R}$ and $m_{0}$ in the circulation model in Equations (11) and (12), allowing us to find (almost) unique solutions after initial transients disappear.

**Table 2:** Typical model parameters. The pathological case is in square brackets.

Parameter	Value	Physiological Meaning
$R_{A}$	0.001 mmHg s/mL	Aortic valve resistance
$R_{M}$	0.005 mmHg s/mL	Mitral valve resistance
$R_{S}$	0.5 mmHg s/mL	Systemic vascular resistance
$R_{C}$	0.0398 mmHg s/mL	Characteristic resistance
$R_{*}$	$0.3061-3.5\;\left(P_{V}-1\;mmHg\right)\;\Theta \left(1\;mmHg-P_{V}\right)$	Total pump resistance
$C_{R}$	4.4 ml/mmHg	Left atrial compliance
$C_{S}$	1.33 ml/mmHg	Systemic compliance
$C_{A}$	0.08 ml/mmHg	Aortic compliance
$L_{S}$	0.0005 mmHg·s^2^/mL	Inertance of blood in aorta
$L_{*}$	0.0472 mmHg·s^2^/mL	Total pump inertance
$\sigma_{0}$	240 kpa	Maximum sarcomere active tension
$k_{0}$	120 kpa	Maximum sarcomere active elastance
$k_{1}$	0.002 kpa	Model parameter for a passive tension
$k_{2}$	14 kpa [40 kpa]	Model parameter for a passive tension
$\chi ,\;\alpha_{l}$	100 s^−1^, 10 m^−1^	Damping parameters in sarcomere
$\omega_{0}^{}$	100 s^−1^	Sarcomere microscale oscillation frequency
a, b	100 m·s^−2^ kpa^−1^, 6000 m·s^−2^	Active and passive force parameters
$\beta_{0}$	20 mL^−2^	Length-tension parameter
γ	0.6 [0.45]	$P_{V}$ parameter
$V_{0}$	144/1.5 mL [144 mL]	Volume parameter
$\mu_{1},\;\mu_{2}$	0.0024 kpa^−1^, 0 (s·mL)^−1^	MEF parameters
